# Comparative assessment of genetic diversity matrices and clustering methods in white Guinea yam (*Dioscorea rotundata*) based on morphological and molecular markers

**DOI:** 10.1038/s41598-020-69925-9

**Published:** 2020-08-06

**Authors:** Kwabena Darkwa, Paterne Agre, Bunmi Olasanmi, Kohtaro Iseki, Ryo Matsumoto, Adrian Powell, Guillaume Bauchet, David De Koeyer, Satoru Muranaka, Patrick Adebola, Robert Asiedu, Ryohei Terauchi, Asrat Asfaw

**Affiliations:** 1 International Institute of Tropical Agriculture (IITA), Ibadan, Nigeria; 2 Institute of Life and Earth Sciences, Pan African University, University of Ibadan, Ibadan, Nigeria; 3 Department of Agronomy, University of Ibadan, Ibadan, Nigeria; 4 Japan International Research Center for Agricultural Sciences, Tsukuba, Japan; 5 Boyce Thompson Institute, Ithaca, NY USA; 6 International Institute of Tropical Agriculture (IITA), Abuja, Nigeria; 7 Iwate Biotechnology Research Center, Kitakami, Iwate Japan; 8 Present Address: Agriculture and Agri-Food Canada, 850 Lincoln Road, Fredericton, NB E3B 4Z7 Canada

**Keywords:** Genetics, Plant sciences

## Abstract

Understanding the diversity and genetic relationships among and within crop germplasm is invaluable for genetic improvement. This study assessed genetic diversity in a panel of 173 *D. rotundata* accessions using joint analysis for 23 morphological traits and 136,429 SNP markers from the whole-genome resequencing platform. Various diversity matrices and clustering methods were evaluated for a comprehensive characterization of genetic diversity in white Guinea yam from West Africa at phenotypic and molecular levels. The translation of the different diversity matrices from the phenotypic and genomic information into distinct groups varied with the hierarchal clustering methods used. Gower distance matrix based on phenotypic data and identity by state (IBS) distance matrix based on SNP data with the UPGMA clustering method found the best fit to dissect the genetic relationship in current set materials. However, the grouping pattern was inconsistent (r = − 0.05) between the morphological and molecular distance matrices due to the non-overlapping information between the two data types. Joint analysis for the phenotypic and molecular information maximized a comprehensive estimate of the actual diversity in the evaluated materials. The results from our study provide valuable insights for measuring quantitative genetic variability for breeding and genetic studies in yam and other root and tuber crops.

## Introduction

Yam (*Dioscorea* spp.) is a widely cultivated crop in the tropics and subtropics for its edible starchy tubers. The crop is, however, most prominent in five countries in West Africa (Nigeria, Ghana, Côte d’Ivoire, Benin, and Togo), known as the “yam belt,” an area accounting for 92% of global yam production^1^. Of the over 600 *Dioscorea* species^2^, *D. rotundata*, native to West Africa, is the most important in terms of volume of production^3^ and the most preferred in the yam belt due to its suitability for many traditional foods^2^. Besides the food and economic value, yam is very important in traditional and contemporary medicine^4,5^ and has social, cultural, and religious relevance in West Africa^6^.

The genetic variability of crops held in gene banks, wild and cultivated varieties as well as elite breeding lines serve as gene pools from which breeders continually source rare alleles of essential traits for introgression into adapted lines and for the generation of new variability for selection. Been the bedrock of plant breeding endeavors without which there would not be much scope for crop improvement, breeders have, over the years, employed many strategies to explore and quantify the extent of variability in plant populations^7^. Genetic diversity in *D. rotundata* has been assessed using morphological traits^8,9^, isozymes^10,11^, amplified fragment length polymorphism^12^, simple sequence repeats^13,14^, random amplified polymorphic DNA^15^ and single nucleotide polymorphisms^16^. As a result of the strong environmental influence on their expression, phenotypic markers may not precisely capture the available diversity in a population^7,17^. Molecular markers, particularly SSR and SNPs have been widely employed to study the diversity in many species with much success, however, very low or negligible correlations have been reported between the dissimilarity matrices from the genotypic and phenotypic data^18,19^. Hence, it suggests that the non-overlapping information is emanating from the phenotypic and genotypic dissimilarity matrices. Combining such information for diversity analysis would provide a comprehensive overview of the total diversity in a population^20–22^. This approach, which seeks to explore the synergistic benefits of morphological and molecular markers in the evaluation of genetic variability and population structure, has not gained much attention in yam.

A standard approach applied to study genetic diversity is the comparison of individual genotypes within and between populations using a genetic dissimilarity matrix of all potential pairwise combinations of individuals for characterizing population structure based on relative affinity of everyone to all other individuals evaluated^23^. Several measures, including Euclidean, Manhattan, Mahalanobis, and Gower coefficient, are frequently employed in the analysis of dissimilarity of individuals using phenotypic attributes. In contrast, other dissimilarity matrices such as Nei, Jaccard, the Identity by state (IBS), and Rogers are applied for molecular markers^24^. These similarity coefficients are defined differently and so may produce different results for both the qualitative and quantitative relationships among individuals^23,24^, hence, the choice of an appropriate similarity index is very crucial for determining actual genetic dissimilarity among individuals. Also, affecting the results of genetic diversity studies is the method used for summarizing the dissimilarity matrices into groups or clusters^25^. Hierarchical clustering is the most widely used approach in the analysis of crop genetic diversity^26^. Several hierarchical clustering methods, including single linkage, complete linkage, simple average, median, unweighted paired group method using arithmetic averages (UPGMA), McQuitty, and Ward’s minimum variance have been used^25,26^. Each of these approaches has some distinctive features and may generate different results, hence, the choice of an appropriate method to meet the desired objectives is very imperative^25^. Comparative studies of different dissimilarity matrices, as well as hierarchical clustering methods, have been conducted to identify the appropriate approach for genetic diversity assessment in many crops, including sweetpotato^18^, switchgrass^21^, and maize^27^, but not yet for white Guinea yam. The objectives of this study were to (1) compare different dissimilarity matrices and hierarchical clustering methods for evaluating genetic diversity in white Guinea yam, (2) assess the genetic diversity and differentiation in a population of white Guinea yam using morphological, molecular and combined data.

## Results

### Principal component analysis

Results of the principal component analysis (PCA) indicated that the first ten components with eigenvalues ranging from 1.01 to 6.26 were important in explaining the variation among the 173 accessions studied and cumulatively accounted for 72.32% of the total phenotypic variation (Table 1). The first principal component (PC) accounted for 20.87% of the total variation. It illustrated the variations in stem diameter, plant vigor, plant sex, tuber yield per plant, tuber yield per hectare, average tuber weight, leaf density, tuber length, and tuber width primarily. Principal component two contributed 11.85% to the total variation. Seven variables, including days to maturity, days to flowering, tuber dry matter content, tuber flesh oxidation, yam mosaic virus severity, and tuber surface cracks, were identified to contribute most to PC two. The third PC emphasized the number of stems and number of tubers per plant and explained 7.55% of the total variation. Principal components 4 and 5 accounted for 5.94% and 5.43% of the total variance and explained the variation in tuber appearance and tuber area, respectively. Out of the 30 traits evaluated, 23 were found to contribute most to the first ten principal components (Table 1) and were therefore considered most discriminant to summarize phenotypic variation among the accessions through hierarchical cluster analysis. Phenotypic variations of the selected 23 variables were assessed (mean, median, minimum, maximal, Kurtosis variation, and standard error) and a summary presented in Supplementary Table S1.

**Table 1:** Eigenvalues, variance, cumulative variance, and principal component scores (Eigenvectors) of the first ten components of genetic divergence in a panel of 173 *D. rotundata* accessions.

Variables	PC1	PC2	PC3	PC4	PC5	PC6	PC7	PC8	PC9	PC10
Days to start senescence	0.21	0.79	− 0.14	0.18	0.15	0.02	− 0.19	0.04	0.11	0.02
Days to flowering	0.06	0.59	0.07	0.15	0.14	− 0.19	0.12	0.24	− 0.15	0.18
Days to maturity	0.29	0.67	− 0.01	0.25	0.04	0.10	− 0.04	0.04	0.11	0.04
Number of stems	− 0.30	0.07	0.72	− 0.09	0.00	0.22	0.01	− 0.19	0.18	− 0.03
Stem diameter	0.51	− 0.24	− 0.26	0.18	− 0.01	− 0.34	− 0.17	0.25	− 0.09	0.32
YAD (AUDPC value)	− 0.21	0.01	0.37	− 0.20	− 0.04	− 0.38	0.09	0.08	− 0.04	0.10
YMV (AUDPC value)	− 0.10	0.78	0.02	0.27	0.10	− 0.21	− 0.20	− 0.06	0.07	0.12
Plant vigor	0.55	− 0.23	0.27	− 0.04	− 0.15	− 0.25	− 0.10	0.04	− 0.16	0.19
Plant sex	0.53	− 0.06	0.20	0.41	0.21	− 0.14	− 0.06	0.10	− 0.01	− 0.52
Flowering intensity	0.48	− 0.10	0.13	0.01	0.00	− 0.20	− 0.47	− 0.17	− 0.32	− 0.49
Number of tubers plant^−1^	− 0.10	0.07	0.78	0.19	− 0.20	0.27	0.12	0.10	− 0.13	0.08
Tuber yield (kg plant^−1^)	0.91	0.01	0.24	− 0.10	− 0.10	0.07	0.08	− 0.02	0.12	0.08
Tuber yield (t ha^−1^)	0.91	0.01	0.23	− 0.09	− 0.09	0.08	0.08	− 0.02	0.12	0.08
Average tuber weight (kg)	0.91	− 0.02	− 0.04	− 0.18	− 0.04	− 0.05	0.02	− 0.06	0.14	0.09
Tuber appearance	0.16	0.40	0.21	− 0.53	− 0.21	0.02	0.02	0.16	− 0.11	− 0.14
Spines on tuber	0.38	− 0.07	0.15	0.33	0.00	0.22	0.01	− 0.48	− 0.10	0.18
Tuber cracks	− 0.28	− 0.53	− 0.08	0.14	− 0.10	− 0.19	0.06	− 0.05	0.37	− 0.01
Tuber hairiness	0.42	− 0.15	− 0.03	0.29	0.41	0.40	0.03	− 0.33	− 0.15	0.05
Canopy architecture	− 0.04	− 0.04	0.43	− 0.19	0.14	0.16	− 0.45	0.42	0.31	− 0.05
Leaf density	0.76	− 0.17	0.21	0.13	− 0.22	0.03	0.22	0.17	− 0.11	0.05
Leaf shape	− 0.38	0.26	− 0.17	− 0.28	0.04	0.38	0.20	0.00	0.02	− 0.21
Senescence class	− 0.42	− 0.02	0.30	− 0.16	0.22	− 0.35	− 0.06	− 0.37	0.07	0.25
Spines on stem	0.18	− 0.22	− 0.17	− 0.18	0.40	0.46	0.00	0.31	− 0.20	0.25
Inflorescence type	0.22	− 0.10	0.05	0.39	0.19	− 0.06	0.34	0.17	0.59	− 0.12
Stem color	− 0.08	0.13	− 0.07	0.19	− 0.37	− 0.14	0.59	0.05	− 0.22	− 0.17
Tuber length	0.65	0.00	− 0.15	− 0.48	0.36	− 0.16	0.21	− 0.13	0.10	− 0.10
Tuber width	0.70	0.03	− 0.34	− 0.28	− 0.29	0.09	− 0.08	− 0.14	0.23	− 0.04
Tuber area	− 0.05	0.00	− 0.26	0.23	− 0.71	0.30	− 0.35	0.02	0.12	0.07
Tuber flesh oxidation	0.28	0.57	− 0.08	− 0.02	0.07	0.04	0.10	0.13	− 0.12	− 0.04
Dry matter content	− 0.04	− 0.65	0.02	0.18	0.21	0.07	− 0.03	0.33	− 0.13	− 0.03
Eigenvalue	6.26	3.56	2.26	1.78	1.63	1.51	1.34	1.21	1.13	1.01
% variance	20.87	11.85	7.55	5.94	5.43	5.05	4.45	4.05	3.76	3.38
Cumulative variance (%)	20.87	32.72	40.27	46.20	51.64	56.68	61.14	65.18	68.94	72.32

*PC* principal component, *YAD* yam anthracnose disease, *YMV* yam mosaic virus, *AUDPC* area under disease progression curve.

### Assessment of diversity matrices and clustering methods for phenotypic and molecular data

Table 2 presents the cophenetic correlation coefficients (CCC) for translating phenotypic and genotypic information from various dissimilarity matrices into a dendrogram using different clustering methods. The translation of various dissimilarity matrices from the phenotypic information into a dendrogram showed consistently higher CCC (> 0.70) with the UPGMA method. Among the four dissimilarity matrices calculated for the phenotypic traits, the Gower distance showed the highest CCC value 0.91 with UPGMA method. The cophenetic correlation coefficients between the various distance matrices of molecular markers and hierarchal clustering methods were generally higher (> 0.79) than that of phenotypic distance matrices. The IBS matrix, however, showed a high correlation with the UPGMA clustering method. The UPGMA method proved superior to the other techniques in translating the information from the combined matrix (Gower + IBS) into a dendrogram too. Based on the cophenetic correlation, employing the IBS and Gower dissimilarity matrices with the UPGMA method was found to be suitable for clustering the accessions based on the genotypic and phenotypic information, respectively.

**Table 2:** Results of the cophenetic correlation coefficients (CCC) for comparing diversity matrices and clustering methods for phenotypic and molecular data in white Guinea yam.

Dissimilarity matrices	Clustering methods					
	Ward.D2	Single	Average (UPGMA)	Median	Mcquitty (WPGM)	Complete
**Phenotypic data**						
Gower	0.58	0.67	0.91	0.61	0.80	0.78
Manhattan	0.74	0.85	0.90	0.81	0.86	0.88
Euclidean	0.74	0.85	0.90	0.81	0.86	0.87
Mahalanobis	0.59	0.83	0.85	0.81	0.84	0.81
**Genotypic data**						
IBS	0.80	0.87	0.91	0.83	0.90	0.88
Jaccard	0.80	0.85	0.90	0.79	0.89	0.86
Nei	0.81	0.87	0.90	0.85	0.89	0.88
Roger	0.81	0.87	0.90	0.85	0.89	0.88
Gower + IBS	0.56	0.62	0.75	0.62	0.67	0.71

### Clustering pattern based on morphological diversity

**Figure 1:**
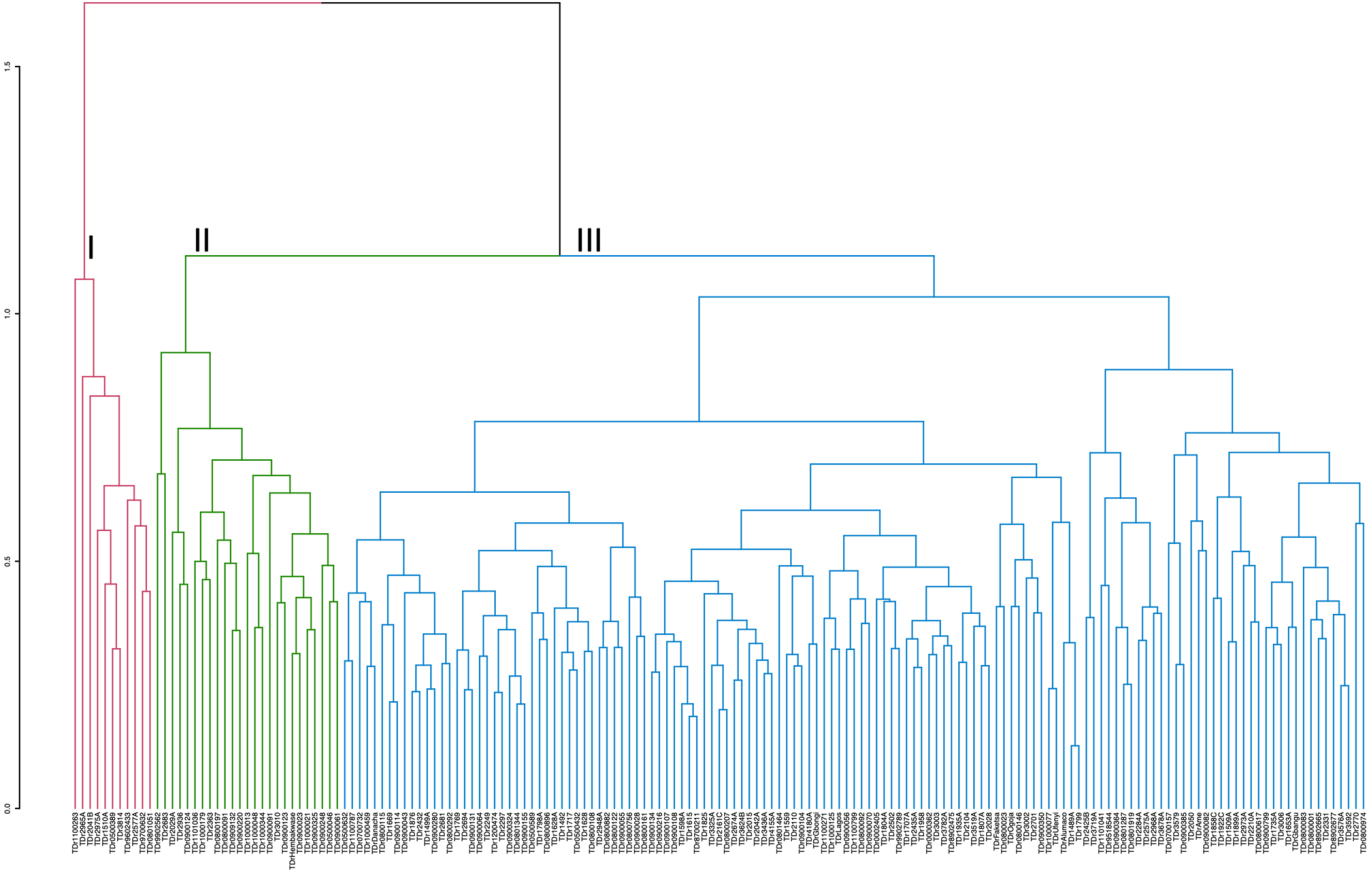
Hierarchical cluster dendrogram based on the ‘Gower’ morphological dissimilarity matrix using the 23 most discriminant phenotypic traits showing the grouping pattern of the 173 *Dioscorea rotundata* accessions evaluated.

The grouping pattern of the 173 *D. rotundata* accessions for morphological diversity using Gower distance in UPGMA method showed three major clusters (Fig. 1). The cluster size varied between groups identified with a larger number of accessions in cluster three (blue) containing 137 accessions (79%), of which 66 were genebank accessions, 62 breeding lines from IITA as well as nine farmers’ varieties. Accessions in cluster three were generally highly susceptible to yam mosaic virus disease, less vigorous, low yielding, and with moderate tuber dry matter content. The second cluster (green) was made up of 25 accessions, out of which 19 were breeding lines, five genebank accessions, and a farmer's variety. Cluster two accessions were high yielding, with longer and broader tubers characterized by high oxidation. In cluster one (red) were 11 accessions, comprising of five breeding lines and six genebank accessions. Accessions in cluster one were early flowering and maturing, tolerant to the YMV disease, and produced tubers with many cracks, high dry matter content, and no oxidation.

### Summary statistics and clustering pattern of accessions based on molecular diversity indices

The minor allele frequency of the 136,429 SNP markers used in this study varied from 0.052 to 0.50, with an average of 0.26 (Supplementary Table S2). The mean observed and expected heterozygosity were 0.42 and 0.35, respectively. Polymorphic information content was high across the SNPs, with an average of 0.28. The mean Hardy Weinberg Equilibrium was 0.20.

Using the IBS dissimilarity matrix, the genetic distance for the entire population varied from 0.05 to 0.31. The genetic distance was highest between TDr2161C (genebank accession from Nigeria) and TDr0900055 (a breeding line from the hybridization of TDr9700973 and TDr9501932), while it was lowest between TDr4180A (landrace from Guinea) and TDr2674A (landrace from Nigeria).

**Figure 2:**
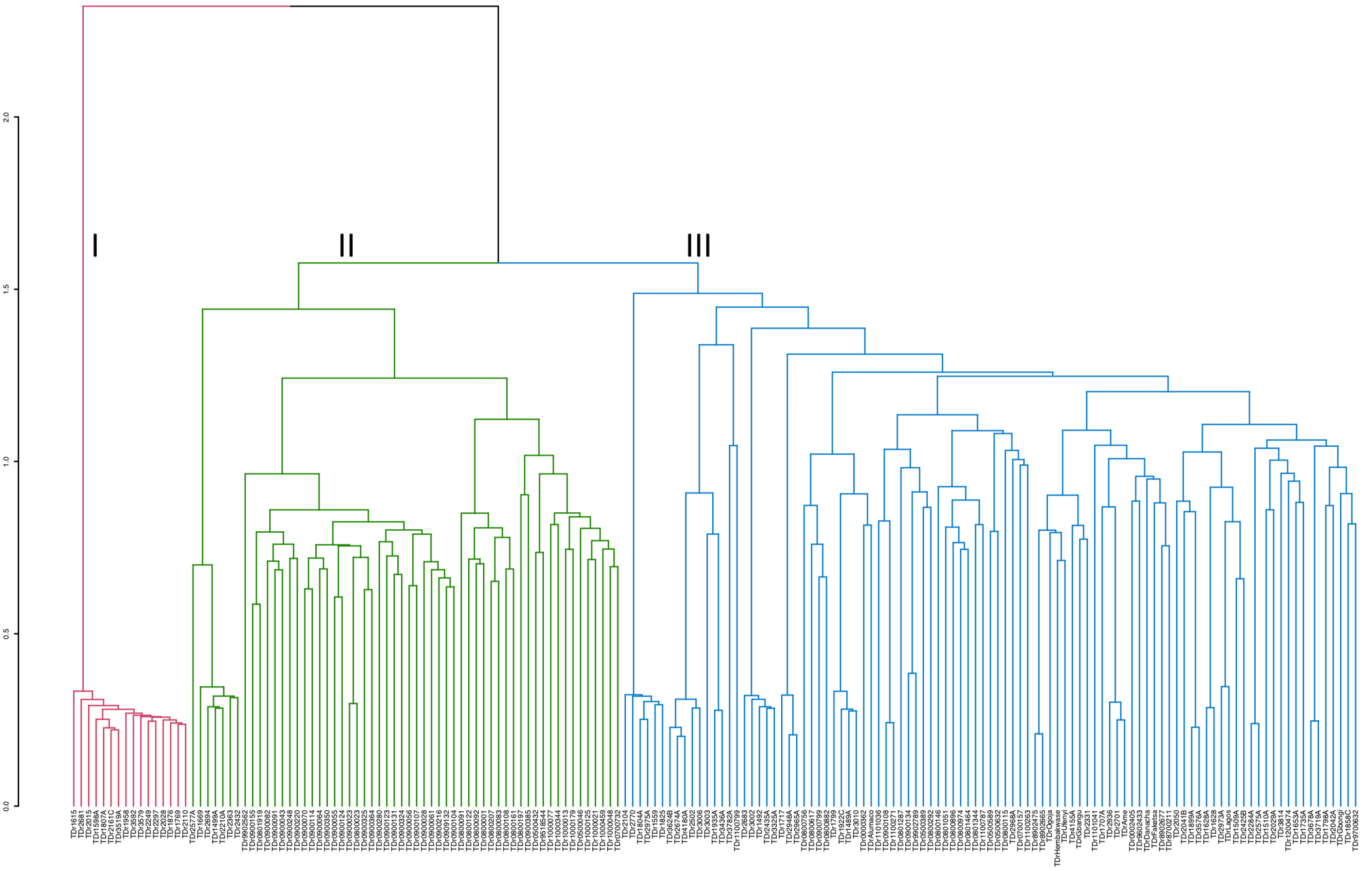
Hierarchical cluster representing the genetic relationships among the 173 *D. rotundata* accessions based on the Identity by state (IBS) dissimilarity matrix obtained from the 136,429 SNP markers. Each color represents a different cluster.

Using the 136,429 SNP markers, the 173 accessions were grouped into three major clusters (Fig. 2). Cluster three (blue) was the biggest with 99 accessions comprising of 54 genebank accessions from six countries with the highest number of accessions from Togo (27) followed by Nigeria (20) (Supplementary Information 1). The third cluster contained in addition to the genebank accessions, 35 breeding lines from IITA, and ten farmers’ varieties from Nigeria. The 35 breeding lines in cluster three were full-sibs and half-sibs from the bi-parental or open pollination of 11 females and ten males (Supplementary Information 1). Cluster two (green) contained 58 accessions, of which 51 were breeding lines, while the remaining seven were genebank accessions collected from Cote d'Ivoire (1), Nigeria (4), and Togo (2). The breeding lines in cluster two originated from bi-parental crosses involving eight females and three males. Out of the 51 breeding lines grouped in cluster two, 35 lines shared the same male parent (TDr9501932) and three female parents (TDr0200076, TDr9518544, and TDr9700973). Cluster one (red) was the smallest group containing 16 accessions, all of which were genebank accessions collected from Benin Republic (1), Cote d'Ivoire (1), Ghana (1), Nigeria (4) and Togo (9) (Supplementary Information 1). Genetic distance in cluster one varied from 0.062 (TDr3002 and TDr1807A) to 0.083 (TDr1615 and TDr3592).

### Genetic diversity indices and grouping

Table 3 presents the most widely used genetic diversity indices, the Shannon–Wiener index, Simpson’s indices, and Pilou evenness index calculated for 173 white Guinea yam accessions based on phenotypic and molecular data. The diversity indices calculated were generally high and did not differ significantly for the phenotypic and molecular data. Similarity in the genetic diversity indices distribution was observed for the phenotypic and molecular data. However, the inverse Simpson’s index was yet higher at the molecular level compared to the phenotypic level.

**Table 3:** Genetic diversity indices based on phenotypic and SNP data in the *D. rotundata* accessions.

	Shannon–Wiener Index (H′)	Inverse Simpson’s (HB)	Simpson's Index (λ)	Pilou evenness (J)	Fixation index (Fst)
Phenotypic	5.11	160.0	0.9937	0.1933	NA
Genotypic	5.14	169.7	0.9941	0.1933	0.15783

**Figure 3:**
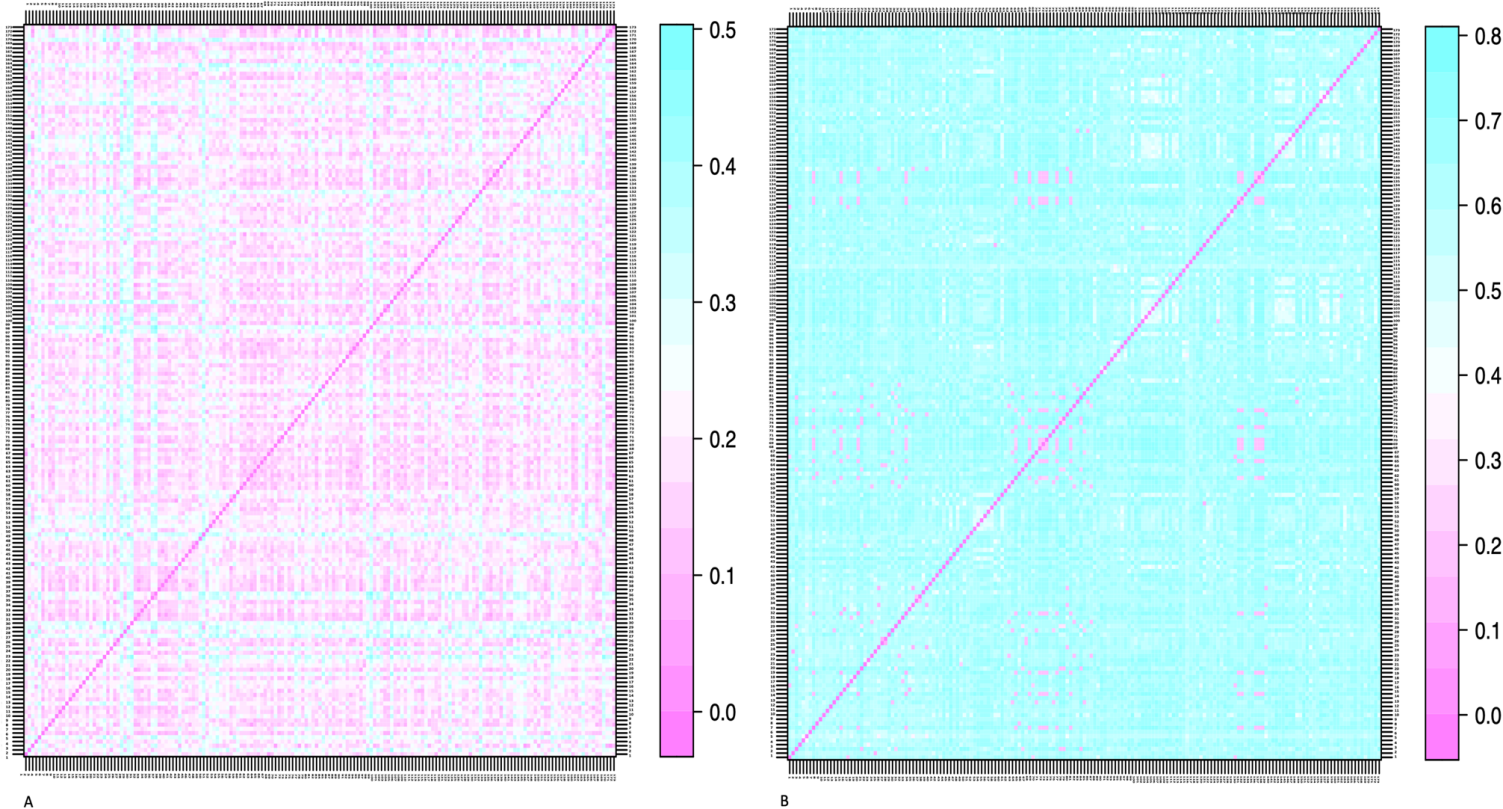
(**A**) Gower’s dissimilarity matrix from the phenotypic data and (**B**) IBS dissimilarity matrix generated from the genotypic data of the *D. rotundata* accessions. The color gradient graphically expresses the dissimilarity between the white yam accessions. Pink indicates the most similar accessions, while the blue color indicates the most dissimilar accessions. The dissimilarity matrices were symmetric, and values below the diagonal are equivalent to those above the diagonal.

**Figure 4:**
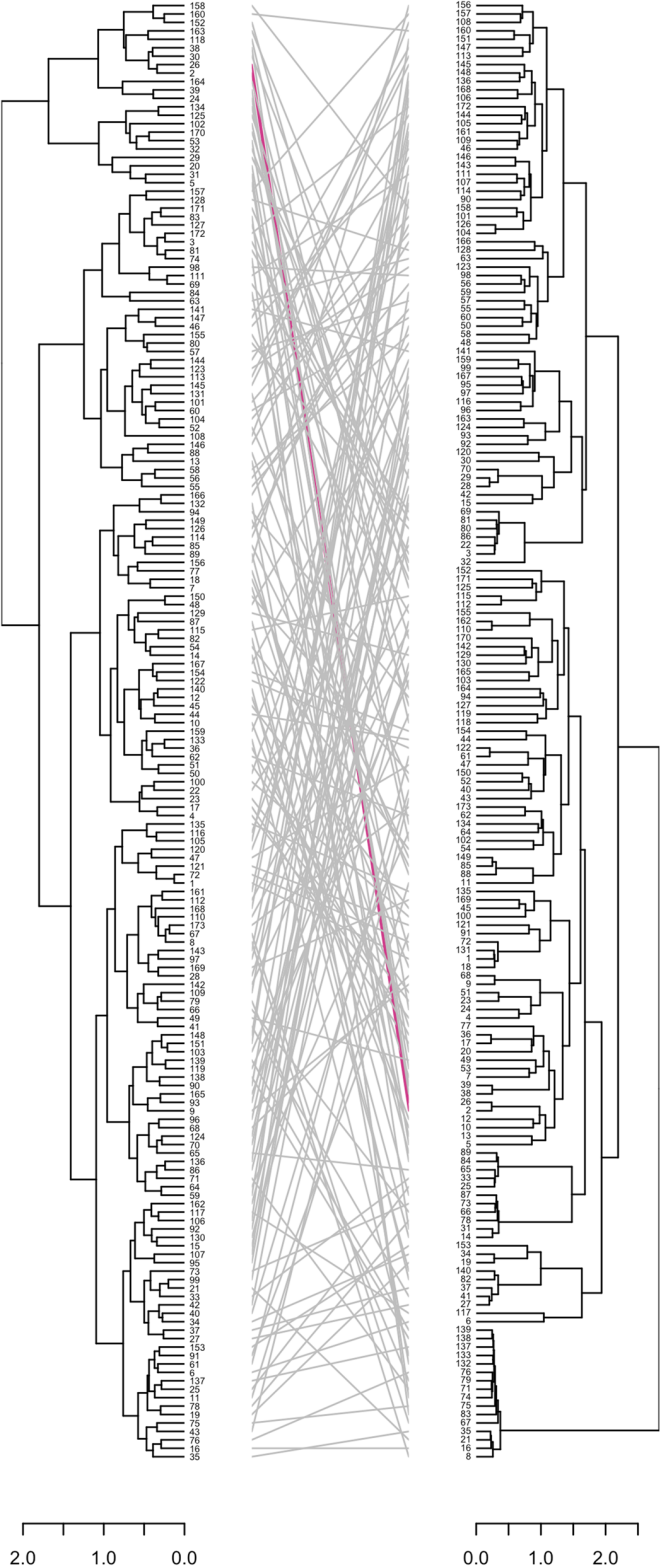
Comparison of hierarchical clustering dendrograms of the 173 *D. rotundata* accessions from phenotypic (left) and the genotypic (right) data. The black lines in between the two dendrograms represent mismatched accessions while the purple lines are accessions in the same position from phenotypic to the genotypic cluster.

Assessment of morphological diversity with Gower distance matrix revealed low variability among the accessions studied, as shown by the copious pink dots in Fig. 3A. Conversely, genetic variation was high among the *D. rotundata* accessions with the dissimilarity matrix emanating from the SNP data, as shown by the high number of blue dots in Fig. 3B. The hierarchical cluster generated from the phenotypic information was compared to that originating from the genotypic data (Fig. 4). Out of the 173 accessions evaluated, only two maintained the same cluster position across the two hierarchical cluster dendrograms (Fig. 4).

### Genetic diversity using joint analysis for morphological and molecular data

**Figure 5:**
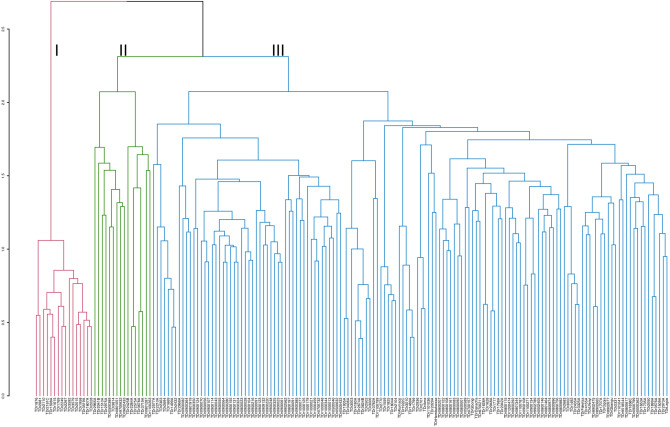
Hierarchical clustering of the 173 *D. rotundata* accessions based on the combined phenotypic (Gower matrix) and molecular data (IBS) using the UPGMA method. Each color represents different cluster.

The 173 *D. rotundata* accessions were partitioned into three distinct clusters using the combined dissimilarity matrix of phenotypic and molecular marker information. Cluster membership ranged from 16 to 141 accessions. Cluster three (blue) was composed of 141 accessions, including 80 breeding lines, 51 genebank accessions, and ten farmers’ varieties (Fig. 5). Cluster two (green) contained 16 clones that included ten genebank accessions and six breeding lines. Cluster one (red) was made up of 16 genebank accessions. Accessions in cluster three generally had higher tuber yielding potential with late flowering, late maturing, high flowering intensity, thicker stems, more prone to yam mosaic virus disease, and low tuber dry matter content (Table 4). Accessions in cluster two were early to flower and mature with negligible tuber flesh oxidation, low tuber yield, and high tuber dry matter content. Accessions in cluster two were also characterized by multiple stems, low flowering intensity, high tuber cracks, and less susceptible to yam mosaic virus disease (compared to those in clusters 1 and 3). For most of the traits evaluated, accessions in cluster one showed moderate performance in comparison to clusters two and three. Accessions categorized in the first cluster had average yield with longer tuber length, broader in size as well as high tuber flesh oxidation.

**Table 4:** Phenotypic and genotypic parameter variation across the genetic groups identified by the combined analysis.

Phenotypic traits	Cluster 1		Cluster 2		Cluster 3	
	Average	SD	Average	SD	Average	SD
Days to start senescence	226.15	10.11	177.98	38.44	229.58	4.43
Days to flower	139.88	41.67	81.03	32.45	136.92	18.27
Days maturity	254.10	22.75	202.82	39.16	249.27	4.41
No. of stems	1.45	0.57	2.12	1.66	1.13	0.16
Stem diameter	3.96	0.77	3.70	1.03	3.47	0.62
YMV (AUDPC value)	350.03	30.53	268.33	86.06	330.13	19.89
Plant vigor	1.83	0.33	1.76	0.52	1.75	0.22
Plant sex	0.93	0.65	0.47	0.62	0.54	0.49
Flower intensity	3.17	2.40	2.21	1.55	2.55	2.54
Number of tubers per plant	1.38	0.45	1.38	0.44	1.08	0.17
Tuber weight (kg plant^−1^)	1.13	0.48	0.69	0.44	1.00	0.35
Tuber weight (t ha^−1^)	11.15	4.68	6.65	4.39	9.88	3.49
Average tuber weight	1.00	0.44	0.63	0.47	0.98	0.37
Tuber appearance	1.85	0.65	1.37	0.61	2.07	0.62
Tuber cracks	0.50	0.45	1.27	1.02	0.23	0.28
Leaf density	5.02	0.72	4.48	1.16	4.76	0.54
Inflorescence type	1.23	0.35	1.16	0.25	1.08	0.10
Stem color	1.64	0.69	1.43	0.48	1.76	0.70
Tuber length	22.34	5.31	18.78	7.10	26.12	2.11
Tuber width	9.43	2.32	7.61	2.19	10.16	2.48
Tuber area	0.44	0.10	0.46	0.18	0.40	0.10
Oxidation	1.75	1.27	0.09	0.85	2.29	1.55
Dry matter	33.61	3.38	37.96	3.38	35.30	4.78
Minor allele frequency	0.26		0.26		0.22	
Observe heterozygosity	0.42		0.43		0.44	
Expected heterozygosity	0.35		0.34		0.25	
Polymorphism information content	0.26		0.65		0.56	

**Figure 6:**
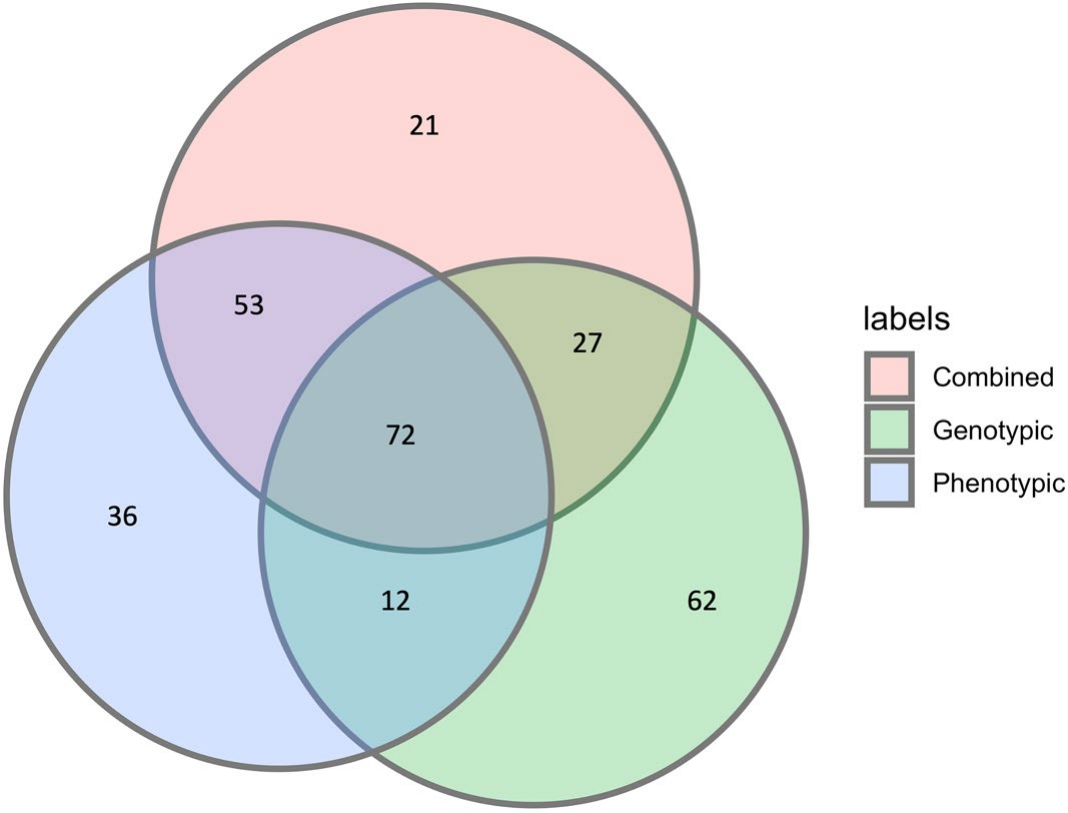
Venn diagram showing the concordance of cluster memberships across the phenotypic, genotypic and combined clusters of the 174 *D. rotundata* accessions.

A comparison of the cluster memberships, however, revealed that 72 accessions (42%) were clustered into the same groups by the three methods (Fig. 6). The genotypic and phenotypic clustering grouped 84 accessions into the same groups. In comparison, 125 accessions were clustered in the same groups by the phenotypic and the combined analysis, and 99 accessions appeared in the same genetic groups across the genotypic and combined clusters (Fig. 6).

Minor allele frequency, as well as the observed and expected heterozygosity, showed very low variation across the three genetic groups identified by the combined analysis (Table 4). In contrast, polymorphism information content showed high variation across the genetic groups.

**Figure 7:**
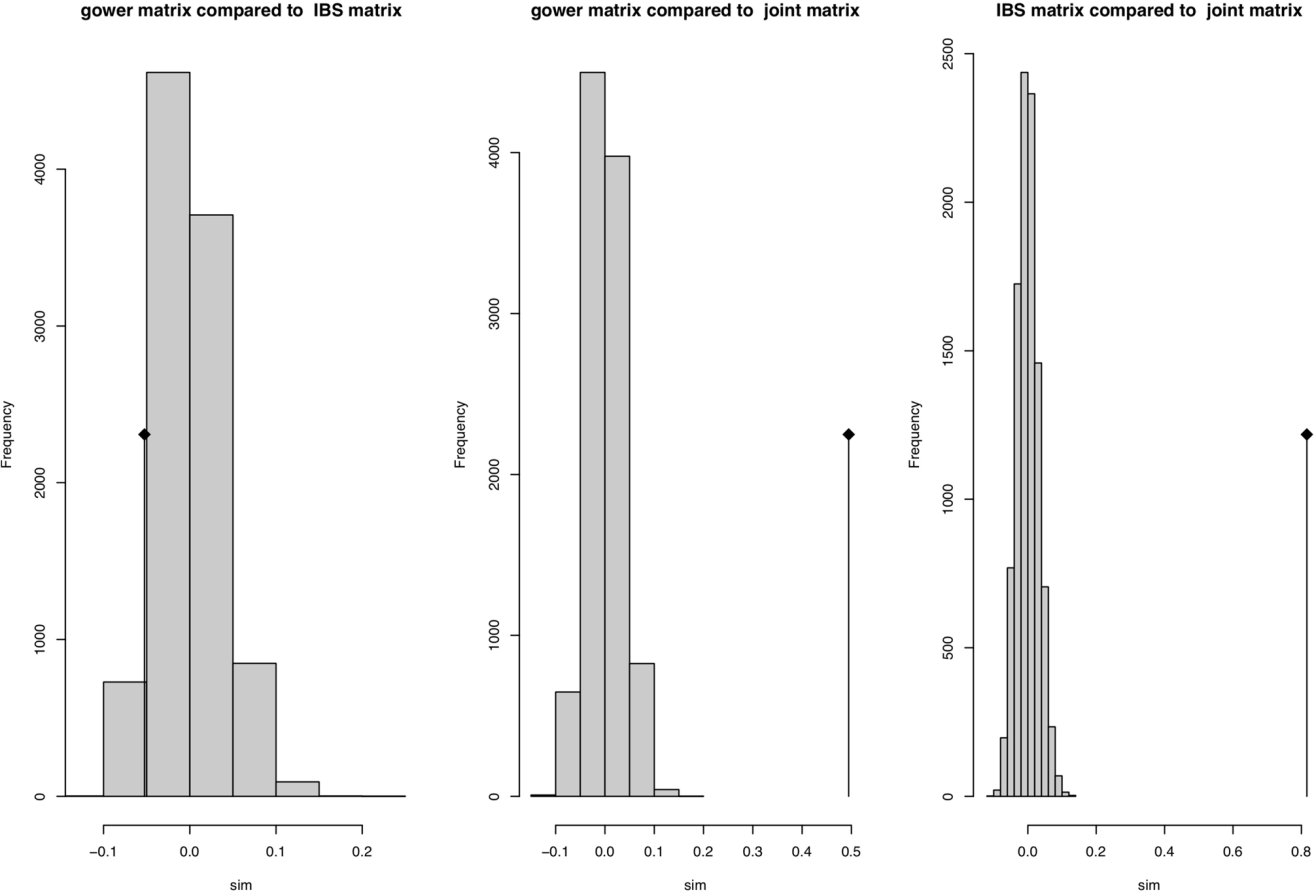
Mantel correlation among phenotypic, genotypic and the combined data.

The Mantel correlation assay between the phenotypic and genotypic dissimilarity matrices was negligible (r = − 0.048) (Fig. 7). However, such correlation was high (r = 0.82) between the genotypic and the combined matrices, and moderate (r = 0.47) between the phenotypic and combined dissimilarity matrices.

## Discussion

Assessment of genetic diversity is an integral aspect of all crop breeding and plant genetic resources management and utilization undertakings; hence, many approaches have been developed to evaluate and quantify the extent of genetic variability in plant populations. This study assessed the variation in a panel of 173 *D. rotundata* accessions using 23 most discriminant morphological traits and 136,429 SNP markers from the whole-genome resequencing genotyping platform. The dissimilarity coefficient, as well as clustering method used for genetic diversity analysis, have implications on the results^23,25^, hence the choice of an appropriate coefficient and hierarchical clustering method is critical for determining the accuracy of the genetic variability among individuals.

High cophenetic correlation coefficients were observed for most of the hierarchical clusters constructed using the different dissimilarity matrices and clustering methods with a few exceptions for both morphological and molecular data. The UPGMA method was observed to give high cophenetic correlation coefficients for most of the dissimilarity matrices across the molecular, morphological, and combined data, demonstrating that there is a good representation of the dissimilarity matrices and distances in the form of dendrograms. The cophenetic correlation coefficient has been widely employed as a measure for evaluating the efficiency of various clustering techniques since its introduction by Sokal and Rohlf^28^ and provides estimates of how precisely a dendrogram preserves the pairwise distances between the original data points^29^. In consonance with the findings of the present study, the UPGMA method of clustering was reported to give high cophenetic correlation coefficients for genetic diversity studies in sweet potato^18^, soyabean^30^, and maize^31^ indicating the viability of using dendrograms from the UPGMA to summarize the information of dissimilarity matrices in genetic diversity studies. Furthermore, Padilla et al.^25^ and Krzanowski^32^ observed high internal affinity within clusters and large variability among clusters generated by the UPGMA compared to the other methods.

Using the 23 morphological traits identified by the first ten principal components as most discriminatory, the UPGMA clustering based on the Gower dissimilarity matrix grouped the 173 white yam accessions into three clusters irrespective of their countries of geographical origin (genebank accessions) and pedigree (breeding lines). Many authors assert that genetic diversity assessment using morphological markers is less reliable due to the strong influence of the environment and plant growth stage on their expression^33–36^. Nevertheless, phenotypic characterization is instrumental in defining the plant population and forms the basis for selecting accessions with desirable traits for crop improvement.

Our analysis of the genetic diversity using the Identity by state (IBS) dissimilarity matrix generated from 136,429 SNP markers partitioned the 173 *D. rotundata* accessions into three groups. The low genetic variability observed among the accessions using the morphological markers could be because variation in phenotypic traits may result from one or few mutations in the genome and epigenetic origin. In contrast, the SNP markers consider variations across the entire genome. The SNP markers, therefore, revealed valuable information about the genetic relationships among the *D. rotundata* accessions enabling the identification of genetically divergent parents helpful for the yam breeding program. Our results suggest the reliability of SNP markers in dissecting the depth of genetic diversity among white Guinea yam accessions, as also reported by Girma et al.^16^ and Scarcelli et al.^37^. Plants showing similar morphological characteristics could be very divergent at the molecular level and vice versa^34,38^. This phenomenon, in addition to the negligible correlation observed between the phenotypic and genotypic dissimilarity matrices in this study, could explain the changing and regrouping observed in comparing the membership of the hierarchical cluster dendrograms emanating from the morphological and molecular characterization. The inconsistency between the clusters identified by the genotypic and phenotypic information could also be attributed to the enormous genotype-by-environment interaction effects generally observed for quantitatively inherited morphological and agronomic traits. The lack of correlation between the molecular and the morphological diversity matrices further emphasizes the non-overlapping and complementarity between the genotypic and the phenotypic information to dissect the nature and extent of genetic diversity in crops^39,40^. Several studies have also reported inconsistencies between phenotypic and genotypic distances in different crops^41–43^.

An approach that combines the phenotypic and genotypic dissimilarity matrices into a single matrix for genetic diversity assessment was suggested to capture the entire genetic variability in plant populations^40,44^. The application of joint analysis for phenotypic and molecular information identified three genetic groups in the current set of materials highly valuable for genetic improvement in white Guinea yam. This has significant implications for yam improvement in that new breeding populations can be developed by hybridization among these three divergent genetic groups, thereby broadening the genetic base of the breeding program. Superior accessions with desirable end-user attributes from these diverse genetic groups are a source of rare alleles for incorporation into elite breeding lines thereby maximizing heterosis in the progenies. The high and moderate correlations observed between the combined matrix and the genotypic and phenotypic dissimilarity matrices, respectively agree with the findings of Alves et al.^19,22^ who dissected genetic diversity in their studies using phenotypic, genotypic and combined distances. These correlations suggest that genetic diversity assessment employing the combined matrix could be an essential tool to capture phenotypic and genotypic information cumulatively. Adequate knowledge about the genetic relationships among accessions is essential to preserve genetic diversity besides identifying superior parental combinations to create segregating populations in a breeding program^45^. Superior clones from the three distinct clusters identified by the combined distances could serve as trait progenitors for hybridization to maximize genetic variability and heterosis in the *D. rotundata* breeding program.

## Conclusion

Genetic diversity analysis of the *D. rotundata* accessions in this study has provided valuable insights to inform breeding strategies and to identify promising parents for the development of improved white Guinea yam varieties with acceptable end-user qualities. High genetic variability was revealed among the white Guinea yam accessions by the SNP makers than the morphological markers, whereas the combined distance showed a high and moderate correlation with the genotypic and phenotypic distances, respectively. Hence quantification of genetic diversity using the combined matrix of phenotypic and genotypic distances explores the synergy of the two approaches, thereby cumulatively capturing the phenotypic and genotypic information to provide a comprehensive outlook of the entire diversity in the population. Clustering of accessions by different dissimilarity coefficients as well as hierarchical clustering methods without careful consideration of these approaches could affect the results. The Gower and IBS dissimilarity matrices presented relatively high cophenetic correlation coefficients using different hierarchical clustering methods; hence, they are more appropriate for genetic diversity studies using phenotypic and genotypic data, respectively.

## Methods

### Plant materials and phenotypic characterization

One hundred and seventy-three *D. rotundata* accessions, including 86 breeding lines, 77 genebank accessions, and ten farmers’ varieties, were used for the study. Details of these accessions, including countries of origin and pedigree information, are provided in Supplementary Information 1. A two-year field experiment was conducted at the experimental field of the International Institute of Tropical Agriculture (IITA/Ibadan Nigeria) (221 m altitude, 07° 29.639″ N, 003° 54.092″ E) during 2017–2018 and 2018–2019 cropping seasons using augmented row–column and randomized block experimental designs, respectively. In 2017–2018, the trial was established with 43 accessions replicated four times and the rest non-replicated in single-row plots of two plants using inter-row and intra-row spacing of one meter in a plot size of 2 m^2^. While, the trial in 2018–2019 was established using a single plant plot arranged in a randomized block design replicated three times. Weeds were controlled manually to keep the experimental field free of weeds all through the growth period of the plants. The accessions were evaluated using thirty agro-morphological traits following the yam crop ontology^46^. The list of traits recorded, period of evaluation and data collection method is summarized in Supplementary Table S3.

### Genotype data

#### DNA extraction and SNP calling

Lyophilized leaves were sent to Iwate Biotechnology Research Center (IBRC-Japan) for DNA extraction, library construction and whole-genome resequencing. For the whole genome sequencing, total genomic DNA was extracted from the leaf samples using a NucleoSpin Plant II Kit according to the manufacturer’s protocol (MachereyNagel GmbH & Co) with slight modifications.

Paired-end sequencing reads generated as fastq files were mapped to the *D. rotundata* reference genome version 2 (https://drive.google.com/drive/folders/1H5T4xjKAEl9LliR-4qK_IR6TypCDe8nj) with Hisat2^47^. The SAM files were converted to BAM format and sorted by name using SAMtools^48^. In cases where multiple sequencing samples were generated from the same biological clone, the corresponding sorted BAM files for each clone were merged using SAMtools. Duplicates were marked and read groups added with the Picard package (https://broadinstitute.github.io/picard/) (v2.17.5). GATK (v3.8-0)^49^ was used to perform indel realignment, variant calling (using HaplotypeCaller in the gVCF mode), and joint genotyping (using GenotypeGVCFs). The VCF file developed was filtered for MAF > 0.1, no missing data both at genotypes and SNP markers level. Only bi-allelic SNP markers with genotype quality > 20, read depth > 5 were retained after using vcftools^50^ and plink^51^ for filtering. The resulting SNPs were subjected to linkage disequilibrium (LD) pruning using the following parameters: 50 bp as window size in SNPs, 5 as step to shift the window and 0.5 as R square and a total of 136,429 SNP markers were retained for all subsequent analysis.

### Data analysis

#### Multivariate analysis of phenotypic data and hierarchical cluster construction

Analysis of variance was performed to determine differences among the accessions for the various traits across the two years using the statistical analysis system software version 9.4^52^ according to the model:

$${Y}_{ijl}=\mu +{B(E)}_{j(l)}+{G}_{i}+{GE}_{ij}+{e}_{ijl}$$

where, *Y* is the trait, µ is the grand mean, E is the environment effect (year), B(E) is the Block effect in environment (year), G is the genotype effect, GE is the genotype by environment interaction, e is the error.

The LSmeans from the genotype by year analysis was used for principal component analysis in the FactorMiner and missMDA R packages^53^. The optimal number of factors to be retained was determined using dimdesc function in R^52^. The selected traits from the above analysis were used in generating four different dissimilarity matrices (Gower, Euclidean, Manhattan and Mahalanobis).

*Gower dissimilarity matrix* was constructed using daisy function in cluster and graphics R packages^54^. Based on this, the dissimilarity matrix was estimated using the following formula:

$${d}_{G}\left({X}_{i}, {X}_{j}\right)=\frac{\sum_{c=1}^{m}{W}_{ijc}{d}_{ijc}}{\sum_{c=1}^{m}{W}_{ijc}}$$

where, ${d}_{ijc}$ is a dissimilarity measure between the i-th and j-th objects by the c-th variable (c = 1, …, m), and ${w}_{ijc}$ takes the value zero, if either the i-th or the j-th object by the c-th variable is missing; otherwise, it takes the value one.

*Euclidean dissimilarity matrix* Euclidean distance was estimated using the Cluster R package^54^ and defined as:

$${\delta }_{eucl}=\sqrt{\sum_{i=1}^{l}({X}_{ip}-{X}_{jp}{)}^{2}}$$

where, *i* and *j* are observations and *p* is the number of variables.

*Manhattan dissimilarity matrix* Manhattan distance, a special case of the Minkowski distance was defined as:

$${d}_{man}= \sum_{i=1}^{n}|{x}_{i}-{y}_{i}|$$

where, x_i_ and y_i_ are two vectors in n-dimensional space.

*Mahalanobis dissimilarity matrix* Mahalanobis distance was estimated according to the formula of Mahalanobis^55^, implemented using the “mahanalobis.dist” function in StatMatch R package^56^. For each variable, the mean and covariance were generated and used as cofactors:

$${d}_{mah}=\sqrt{\left(x-y\right){S}^{-1}(x-y{)}^{T}}$$

where, S is the covariance matrix of the dataset and x and y are two vectors.

### Analysis of molecular markers

SNP marker data of the 173 white yam accessions were used to generate four dissimilarity matrices as follows:

*Identity by State dissimilarity matrix* was generated according to the formula of Wessel and Schork^57^ using tassel software^58^ and converted into a matrix using as.matrix function in R.

$${S}_{i,j}^{IBS}= \frac{\sum_{l=1}^{L}{S}_{i,j}^{l}({g}_{i}^{l},{g}_{j}^{l})}{2L} ,$$

where, *L* is the number of loci considered; ${g}_{i}^{l}$ and ${g}_{j}^{l}$ are the genotypes of individuals *i* and *j,* respectively, at the *j l*th locus (*l* = 1, …, *L*); and ${S}_{i,j}^{l}\left({g}_{i}^{l},{g}_{j}^{l}\right)$ is a function mapping the genotype information for individuals *i* and *j* at locus *l.*

*Nei dissimilarity matrix* was determined by the formula of Nei^59^ using the nei.dist function implemented in poppr R package version 2.8.3^60^

${H}_{s}=\frac{1}{k}\cdot \sum_{s=1}^{k}{H}_{Ss}=\frac{1}{k}\cdot \sum_{s=1}^{k}[1-{q}_{s}^{2}-(1-{q}_{s}{)}^{2}]$ where, k = the total number of loci, H_Ss_ = $1-{q}_{s}^{2}-(1-{q}_{s}{)}^{2}$, and q_2_ is the frequency of one of the two alleles at the *s*th diallelic locus.

*Jaccard dissimilarity matrix* The raw vcf file with the total number of SNPs was converted to the dosage numeric format using plink^51^ and submitted to philentropy R package^61^ to estimate the Jaccard dissimilarity matrix through the following formula:

$$d=1- \frac{\sum_{i=1}^{n}{P}_{i}.{Q}_{i}}{\sum_{i=1}^{n}{P}_{i}^{2}+\sum_{i=1}^{n}{Q}_{i}^{2}-\sum_{i=1}^{n}{P}_{i} . {Q}_{i}}$$

where, n is the total number of elements *i* in P*i* and Q*i*.

*Modified Rogers dissimilarity matrix* was estimated in the cluster R package according to the relation of Rogers^62^:

$${d}_{R}=\frac{1}{m}\sum_{i=1}^{m}\sqrt{\frac{1}{2}\sum_{j=1}^{ni}({p}_{ij}-{q}_{ij}{)}^{2}}$$

where, P_ij_ and q_ij_ are allele frequencies of the *j*th allele at the *i*th locus in the two taxonomic units under consideration, n_i_ is the number of alleles at the *i*th locus, and *m* is the number of loci.

To estimate the correlation between the underlying distance matrix and the distance between instances in the dendrogram using the different dissimilarity matrix, the cophenetic correlation coefficient was estimated^28^ for the different hierarchical clustering methods including ward.D2, single, average (UPGMA), median, McQuitty and complete. Dissimilarity matrix and the hierarchical clustering method with the highest cophenetic correlation coefficient value was retained to plot the final hierarchical cluster dendrogram. Using the method of Alves et al.^22^, graphic representations of the dissimilarity matrices (phenotypic and genotypic) were generated based on color gradients for the expression of dissimilarity among the accessions. A Venn diagram was constructed to assess the agreement of cluster memberships assigned by the phenotypic, genotypic and the combined data. To assess the resemblance between the genotypic and phenotypic matrices and between the genetic dissimilarity matrices and joint dissimilarity matrix, the correlations and their significances were tested with the Mantel Z test with 9,999 permutations using the ade4 R package^63^. Additionally, the Shannon Wiener Index (H′), Inverse Simpson’s (H_B_), Simpson’s Index (λ) and Pilou evenness (J) were assessed using library vegan^64^, while the fixation index (Fst) was assessed using Weir and Cockerham Fst estimates function implemented in vcftools^50^.

## Supplementary information

Supplementary Information.

Supplementary Table S1.

Supplementary Table S2.

Supplementary Table S3.
