# Supplementary material for: Comparative assessment of genetic diversity matrices and clustering methods in white Guinea yam (Dioscorea rotundata) based on morphological and molecular markers
Source: Sci Rep. 2020 Aug 6;10:13191. doi: 10.1038/s41598-020-69925-9 (PMC7413250; doi:10.1038/s41598-020-69925-9)
Supplement: Supplementary file 1 — Supplementary Information. [file 41598_2020_69925_MOESM1_ESM.pdf]

Comparative assessment of genetic diversity matrices and clustering methods in white Guinea yam (*Dioscorea rotundata*) based on morphological and molecular markers

Kwabena Darkwa[1,2], Paterne Agre[1], Bunmi Olasanmi[3], Iseki Kotharo[4], Ryo Matsumoto[1], Adrian Powell[5], Guillaume Bauchet[5], David De Koeyer[6], Satoru Muranaka[4], Patrick Adebola[7], Robert Asiedu[1], Ryohei Terauchi[8], Asrat Asfaw[1,*]

[1]International Institute of Tropical Agriculture (IITA), Ibadan, Nigeria
[2]Pan African University, Institute of Life and Earth Sciences, University of Ibadan, Ibadan, Nigeria
[3]Department of Agronomy, University of Ibadan, Ibadan, Nigeria
[4]Japan International Research Center for Agricultural Sciences, Tsukuba, Japan
[5]Boyce Thompson Institute, Cornell University, Ithaca, New York, USA
[6]Agriculture and Agri-Food Canada, (Current address), 850 Lincoln Road, Fredericton, New Brunswick E3B 4Z7, Canada
[7]International Institute of Tropical Agriculture (IITA), Abuja, Nigeria
[8]Iwate Biotechnology Research Center, Kitakami, Japan

*corresponding author: A.Amele@cgiar.org

Code in Fig. 3 & 4	Clone name	Status	Country origin	Female parent	Male parent	Cluster based on		
						Phenotype	genotype	combined
91	TDr0000362	Breeding line	NA	TDr95/05576	TDr98/00062	3	3	3
54	TDr0002405	Breeding line	NA	NA	NA	3	3	3
92	TDr0500589	Breeding line	NA	TDr95/19177 OP		3	3	3
93	TDr0500632	Breeding line	NA	TDr95/19177 OP		3	3	3
94	TDr0700157	Breeding line	NA	NA	NA	3	3	3
127	TDr0800115	Breeding line	NA	TDr02/00076	TDr95/01932	3	3	3
120	TDr0800146	Breeding line	NA	TDr02/00076	TDr95/01932	3	3	3
171	TDr0800292	Breeding line	NA	TDr02/00076	TDr95/01932	3	3	3
100	TDr0800617	Breeding line	NA	TDr97/00917	TDr96/01818	3	3	3
135	TDr0800756	Breeding line	NA	TDr97/00917	TDr00/00380	3	3	3
169	TDr0800882	Breeding line	NA	TDr97/00917	TDr00/00380	3	3	3
130	TDr0800896	Breeding line	NA	TDr97/00917	TDr99/02626	3	3	3
129	TDr0800974	Breeding line	NA	TDr97/00917	TDr99/02626	3	3	3
103	TDr0801344	Breeding line	NA	TDr02/00076	TDr99/2789	3	3	3
142	TDr0801464	Breeding line	NA	NA	NA	3	3	3
110	TDr0900108	Breeding line	NA	TDr97/00973	TDr95/01932	3	3	3
112	TDr0900134	Breeding line	NA	TDr97/00973	TDr95/01932	3	3	3
45	TDr0900799	Breeding line	NA	NA	NA	3	3	3
162	TDr1100271	Breeding line	NA	TDr97/00205	TDr99/02626	3	3	3
165	TDr1100787	Breeding line	NA	TDr96/00604	TDr1892	3	3	3
117	TDr1100799	Breeding line	NA	TDr96/00604	TDr1892	3	3	3
118	TDr1101041	Breeding line	NA	TDr95/19177	TDr99/02607	3	3	3
119	TDr1200474	Breeding line	NA	TDr96/00634 op		3	3	3
173	TDr8700211	Breeding line	NA	NA	NA	3	3	3
61	TDr8902475	Breeding line	NA	NA	NA	3	3	3
122	TDr8902665	Breeding line	NA	NA	NA	3	3	3
62	TDr8902677	Breeding line	NA	NA	NA	3	3	3

115	TDr9902789	Breeding line	NA	TDr93-50	TDr95/02026	3	3	3
121	TDrAlumaco	Farmer variety	Nigeria	NA	NA	3	3	3
149	TDrAme	Farmer variety	Nigeria	NA	NA	3	3	3
64	TDrDanacha	Farmer variety	Nigeria	NA	NA	3	3	3
43	TDrFaketsa	Farmer variety	Nigeria	NA	NA	3	3	3
44	TDrGbangu	Farmer variety	Nigeria	NA	NA	3	3	3
49	TDrGbongi	Farmer variety	Nigeria	NA	NA	3	3	3
51	TDrLagos	Farmer variety	Nigeria	NA	NA	3	3	3
47	TDrOgoja	Farmer variety	Nigeria	NA	NA	3	3	3
150	TDrUfenyi	Farmer variety	Nigeria	NA	NA	3	3	3
1	TDr1489A	Genebank accession	Togo	NA	NA	3	3	3
65	TDr1492	Genebank accession	Togo	NA	NA	3	3	3
4	TDr1509A	Genebank accession	Togo	NA	NA	3	3	3
66	TDr1559	Genebank accession	Togo	NA	NA	3	3	3
68	TDr1628	Genebank accession	Togo	NA	NA	3	3	3
9	TDr1628A	Genebank accession	Togo	NA	NA	3	3	3
10	TDr1653A	Genebank accession	Togo	NA	NA	3	3	3
11	TDr1707A	Genebank accession	Togo	NA	NA	3	3	3
70	TDr1717	Genebank accession	Togo	NA	NA	3	3	3
12	TDr1735A	Genebank accession	Togo	NA	NA	3	3	3
15	TDr1798A	Genebank accession	Togo	NA	NA	3	3	3
72	TDr1799	Genebank accession	Togo	NA	NA	3	3	3
14	TDr1804A	Genebank accession	Togo	NA	NA	3	3	3
73	TDr1825	Genebank accession	Togo	NA	NA	3	3	3

7	TDr1858C	Genebank accession	Togo	NA	NA	3	3	3
17	TDr1899A	Genebank accession	Benin	NA	NA	3	3	3
18	TDr1922C	Genebank accession	Cote d'Ivoire	NA	NA	3	3	3
19	TDr1935A	Genebank accession	Benin	NA	NA	3	3	3
42	TDr2042A	Genebank accession	Nigeria	NA	NA	3	3	3
77	TDr2050	Genebank accession	Nigeria	NA	NA	3	3	3
78	TDr2104	Genebank accession	Nigeria	NA	NA	3	3	3
154	TDr2331	Genebank accession	Nigeria	NA	NA	3	3	3
25	TDr2435A	Genebank accession	Nigeria	NA	NA	3	3	3
82	TDr2502	Genebank accession	Togo	NA	NA	3	3	3
27	TDr2674A	Genebank accession	Nigeria	NA	NA	3	3	3
85	TDr2701	Genebank accession	Nigeria	NA	NA	3	3	3
87	TDr2770	Genebank accession	Nigeria	NA	NA	3	3	3
28	TDr2948A	Genebank accession	Togo	NA	NA	3	3	3
30	TDr2968A	Genebank accession	Togo	NA	NA	3	3	3
23	TDr2973A	Genebank accession	Togo	NA	NA	3	3	3
89	TDr3002	Genebank accession	Nigeria	NA	NA	3	3	3
153	TDr3003	Genebank accession	Nigeria	NA	NA	3	3	3
140	TDr3006	Genebank accession	Nigeria	NA	NA	3	3	3
33	TDr3325A	Genebank accession	Ghana	NA	NA	3	3	3
34	TDr3436A	Genebank accession	Togo	NA	NA	3	3	3
36	TDr3576A	Genebank accession	Nigeria	NA	NA	3	3	3

37	TDr3624B	Genebank accession	Nigeria	NA	NA	3	3	3
6	TDr3782A	Genebank accession	Benin	NA	NA	3	3	3
40	TDr4155A	Genebank accession	Nigeria	NA	NA	3	3	3
41	TDr4180A	Genebank accession	Guinea	NA	NA	3	3	3
155	TDr1101036	Breeding line	NA	NA	NA	2	3	3
52	TDrHembakwase	Farmer variety	Nigeria	NA	NA	2	3	3
13	TDr2029A	Genebank accession	Nigeria	NA	NA	2	3	3
84	TDr2683	Genebank accession	Nigeria	NA	NA	2	3	3
88	TDr2936	Genebank accession	Togo	NA	NA	2	3	3
131	TDr3010	Genebank accession	Togo	NA	NA	2	3	3
124	TDr0500432	Breeding line	NA	TDr95/19156 op		3	2	3
98	TDr0700732	Breeding line	NA	TDrAmula OP		3	2	3
167	TDr0800001	Breeding line	NA	TDr02/00076	TDr95/01932	3	2	3
126	TDr0800023	Breeding line	NA	TDr02/00076	TDr95/01932	3	2	3
159	TDr0800083	Breeding line	NA	TDr02/00076	TDr95/01932	3	2	3
95	TDr0800092	Breeding line	NA	TDr02/00076	TDr95/01932	3	2	3
96	TDr0800108	Breeding line	NA	TDr02/00076	TDr95/01932	3	2	3
97	TDr0800122	Breeding line	NA	TDr02/00076	TDr95/01932	3	2	3
116	TDr0800161	Breeding line	NA	TDr02/00076	TDr95/01932	3	2	3
99	TDr0800207	Breeding line	NA	TDr02/00076	TDr95/01932	3	2	3
160	TDr0801919	Breeding line	NA	TDr97/00973	TDr95/01932	3	2	3
105	TDr0900028	Breeding line	NA	TDr97/00973	TDr95/01932	3	2	3
157	TDr0900043	Breeding line	NA	TDr97/00973	TDr95/01932	3	2	3
143	TDr0900055	Breeding line	NA	TDr97/00973	TDr95/01932	3	2	3
106	TDr0900056	Breeding line	NA	TDr97/00973	TDr95/01932	3	2	3
90	TDr0900064	Breeding line	NA	NA	NA	3	2	3

107	TDr0900070	Breeding line	NA	TDr97/00973	TDr95/01932	3	2	3
156	TDr0900082	Breeding line	NA	TDr97/00973	TDr95/01932	3	2	3
109	TDr0900104	Breeding line	NA	TDr97/00973	TDr95/01932	3	2	3
168	TDr0900107	Breeding line	NA	TDr97/00973	TDr95/01932	3	2	3
111	TDr0900114	Breeding line	NA	NA	NA	3	2	3
136	TDr0900131	Breeding line	NA	TDr97/00973	TDr95/01932	3	2	3
151	TDr0900155	Breeding line	NA	TDr97/00973	TDr95/01932	3	2	3
161	TDr0900216	Breeding line	NA	NA	NA	3	2	3
172	TDr0900280	Breeding line	NA	TDr97/00973	TDr95/01932	3	2	3
148	TDr0900324	Breeding line	NA	TDr97/00973	TDr95/01932	3	2	3
114	TDr0900350	Breeding line	NA	NA	NA	3	2	3
158	TDr0900364	Breeding line	NA	TDr97/00973	TDr95/01932	3	2	3
166	TDr0900385	Breeding line	NA	NA	NA	3	2	3
48	TDr1000077	Breeding line	NA	TDr9518544	TDr9501932	3	2	3
50	TDr1000125	Breeding line	NA	NA	NA	3	2	3
59	TDr1000459	Breeding line	NA	TDr95/18544	TDr95/01932	3	2	3
163	TDr9518544	Breeding line	NA	TDr87/00571 OP		3	2	3
3	TDr1499A	Genebank accession	Togo	NA	NA	3	2	3
69	TDr1669	Genebank accession	Togo	NA	NA	3	2	3
22	TDr2210A	Genebank accession	Cote d'Ivoire	NA	NA	3	2	3
81	TDr2432	Genebank accession	Nigeria	NA	NA	3	2	3
86	TDr2694	Genebank accession	Nigeria	NA	NA	3	2	3
32	TDr2577A	Genebank accession	Nigeria	NA	NA	1	2	3
123	TDr0500046	Breeding line	NA	TDr02/00410 OP		2	2	3
141	TDr0800091	Breeding line	NA	TDr02/00076	TDr95/01932	2	2	3
128	TDr0800197	Breeding line	NA	TDr02/00076	TDr95/01932	2	2	3
104	TDr0900023	Breeding line	NA	TDr97/00973	TDr95/01932	2	2	3

144	TDr0900061	Breeding line	NA	TDr97/00973	TDr95/01932	2	2	3
108	TDr0900091	Breeding line	NA	TDr97/00973	TDr95/01932	2	2	3
145	TDr0900123	Breeding line	NA	TDr97/00973	TDr95/01932	2	2	3
146	TDr0900124	Breeding line	NA	TDr97/00973	TDr95/01932	2	2	3
147	TDr0900220	Breeding line	NA	TDr97/00973	TDr95/01932	2	2	3
113	TDr0900248	Breeding line	NA	TDr97/00973	TDr95/01932	2	2	3
101	TDr0900325	Breeding line	NA	TDr97/00973	TDr95/01932	2	2	3
46	TDr0909132	Breeding line	NA	NA	NA	2	2	3
55	TDr1000013	Breeding line	NA	TDr9518544	TDr9902789	2	2	3
60	TDr1000021	Breeding line	NA	TDr95/18544	TDr95/01932	2	2	3
56	TDr1000048	Breeding line	NA	TDr9518544	TDr9501932	2	2	3
57	TDr1000179	Breeding line	NA	TDr9518544	TDr9902789	2	2	3
58	TDr1000344	Breeding line	NA	TDr95/18544	TDr95/01932	2	2	3
63	TDr9902562	Breeding line	NA	TDr93-1	TDr95/00858	2	2	3
80	TDr2363	Genebank accession	Nigeria	NA	NA	2	2	3
152	TDr0801287	Breeding line	NA	TDr02/00076	TDr99/2789	3	3	2
2	TDr2284A	Genebank accession	Nigeria	NA	NA	3	3	2
24	TDr2425B	Genebank accession	Togo	NA	NA	3	3	2
26	TDr2575A	Genebank accession	Togo	NA	NA	3	3	2
38	TDr3678A	Genebank accession	Nigeria	NA	NA	3	3	2
39	TDr3719A	Genebank accession	Nigeria	NA	NA	3	3	2
125	TDr0500389	Breeding line	NA	TDr95/19156 op		1	3	2
170	TDr0801051	Breeding line	NA	TDr97/00917	TDr99/02626	1	3	2
164	TDr1100263	Breeding line	NA	TDr97/00205	TDr99/02626	1	3	2
102	TDr9602433	Breeding line	NA	NA	NA	1	3	2
53	TDr9700632	Breeding line	NA	TDr93:24	TDr8902494	1	3	2
5	TDr1510A	Genebank accession	Togo	NA	NA	1	3	2

20	TDr2041B	Genebank accession	Nigeria	NA	NA	1	3	2
29	TDr2965A	Genebank accession	Togo	NA	NA	1	3	2
31	TDr2975A	Genebank accession	Togo	NA	NA	1	3	2
134	TDr3814	Genebank accession	Benin	NA	NA	1	3	2
8	TDr1598A	Genebank accession	Togo	NA	NA	3	1	1
67	TDr1615	Genebank accession	Togo	NA	NA	3	1	1
71	TDr1769	Genebank accession	Togo	NA	NA	3	1	1
16	TDr1807A	Genebank accession	Togo	NA	NA	3	1	1
74	TDr1876	Genebank accession	Benin	NA	NA	3	1	1
137	TDr1958	Genebank accession	Ghana	NA	NA	3	1	1
75	TDr2015	Genebank accession	Togo	NA	NA	3	1	1
76	TDr2028	Genebank accession	Togo	NA	NA	3	1	1
79	TDr2110	Genebank accession	Nigeria	NA	NA	3	1	1
21	TDr2161C	Genebank accession	Nigeria	NA	NA	3	1	1
138	TDr2249	Genebank accession	Togo	NA	NA	3	1	1
139	TDr2297	Genebank accession	Cote d'Ivoire	NA	NA	3	1	1
83	TDr2681	Genebank accession	Togo	NA	NA	3	1	1
35	TDr3519A	Genebank accession	Togo	NA	NA	3	1	1
132	TDr3579	Genebank accession	Nigeria	NA	NA	3	1	1
133	TDr3592	Genebank accession	Nigeria	NA	NA	3	1	1

Note: NA= Not available
