# Supplementary material for: Comparative assessment of genetic diversity matrices and clustering methods in white Guinea yam (Dioscorea rotundata) based on morphological and molecular markers
Source: Sci Rep. 2020 Aug 6;10:13191. doi: 10.1038/s41598-020-69925-9 (PMC7413250; doi:10.1038/s41598-020-69925-9)
Supplement: Supplementary file 2 — Supplementary Table S1. [file 41598_2020_69925_MOESM2_ESM.docx]

| Traits | Mean | Standard Error | Median | Minimum | Maximum | Kurtosis |
| --- | --- | --- | --- | --- | --- | --- |
| Days to start senescence | 222.01 | 1.55 | 227.10 | 133.80 | 239.40 | 10.07 |
| Days to flowering | 134.17 | 3.25 | 134.90 | 44.60 | 288.00 | 1.74 |
| Days to maturity | 248.91 | 2.12 | 251.60 | 153.90 | 337.00 | 4.19 |
| Number of stems per plant | 1.48 | 0.06 | 1.25 | 0.91 | 6.58 | 14.74 |
| Stem diameter | 3.89 | 0.06 | 3.91 | 2.09 | 6.41 | 0.25 |
| YMV (AUDPC value) | 340.64 | 3.41 | 346.27 | 184.42 | 463.43 | 4.96 |
| Plant vigor | 1.82 | 0.03 | 1.83 | 0.94 | 3.04 | 1.65 |
| Plant sex | 0.85 | 0.05 | 1.00 | 0.00 | 3.00 | 0.62 |
| Flowering intensity | 3.02 | 0.18 | 2.50 | 0.00 | 9.00 | -0.83 |
| Number of tubers per plant | 1.35 | 0.03 | 1.17 | 0.97 | 3.18 | 2.49 |
| Tuber yield (kg plant^-1^) | 1.08 | 0.04 | 1.03 | 0.12 | 2.62 | -0.31 |
| Tuber yield (t ha^-1^) | 10.62 | 0.36 | 10.16 | 0.90 | 26.06 | -0.23 |
| Average tuber weight (kg) | 0.96 | 0.03 | 0.92 | 0.04 | 2.41 | -0.36 |
| Tuber appearance | 1.83 | 0.05 | 1.50 | 1.00 | 4.00 | 1.53 |
| Tuber cracks | 0.55 | 0.04 | 0.50 | 0.00 | 3.00 | 4.48 |
| Leaf density | 4.95 | 0.06 | 5.00 | 3.00 | 7.00 | 0.50 |
| Inflorescence type | 1.21 | 0.02 | 1.08 | 1.00 | 2.00 | 1.54 |
| Stem color | 1.63 | 0.05 | 1.75 | 1.00 | 4.00 | -0.05 |
| Tuber length | 22.36 | 0.42 | 22.95 | 5.76 | 36.85 | -0.13 |
| Tuber width | 9.33 | 0.18 | 9.09 | 3.69 | 16.33 | -0.31 |
| Tuber area | 0.44 | 0.01 | 0.42 | 0.21 | 0.92 | 1.91 |
| Tuber flesh oxidation | 1.64 | 0.10 | 1.00 | -1.16 | 5.00 | 1.09 |
| Tuber dry matter (%) | 34.17 | 0.28 | 34.10 | 20.13 | 42.20 | 0.89 |

Supplementary Table S1: Summary statistics of the 23 phenotypic traits of the 173 *D. rotundata* accessions
