# Supplementary material for: Comparative assessment of genetic diversity matrices and clustering methods in white Guinea yam (Dioscorea rotundata) based on morphological and molecular markers
Source: Sci Rep. 2020 Aug 6;10:13191. doi: 10.1038/s41598-020-69925-9 (PMC7413250; doi:10.1038/s41598-020-69925-9)
Supplement: Supplementary file 3 — Supplementary Table S2. [file 41598_2020_69925_MOESM3_ESM.docx]

| Parameter | Min | Max | Average |
| --- | --- | --- | --- |
| Observed heterozygosity | 0.0347 | 1.0000 | 0.4270 |
| Expected heterozygosity | 0.0986 | 0.5000 | 0.3528 |
| Minor allele frequency | 0.0520 | 0.5000 | 0.2600 |
| Polymorphism information content | 0.0930 | 0.3740 | 0.2840 |
| Hardy-Weinberg equilibrium | 1.95E-51 | 1.0000 | 0.2042 |

Supplementary Table S2: Summary statistics of SNP markers
