# Supplementary material for: Comparative assessment of genetic diversity matrices and clustering methods in white Guinea yam (Dioscorea rotundata) based on morphological and molecular markers
Source: Sci Rep. 2020 Aug 6;10:13191. doi: 10.1038/s41598-020-69925-9 (PMC7413250; doi:10.1038/s41598-020-69925-9)
Supplement: Supplementary file 4 — Supplementary Table S3. [file 41598_2020_69925_MOESM4_ESM.docx]

**Supplementary Table S3**: List of phenotypic variables assessed for the diversity analysis of the 173 *D. rotundata* accessions.

| **S/N** | **Variables** | **Collection time** | **Method Used** |
| --- | --- | --- | --- |
| 1 | Days to start senescence | 5 months after planting | number of calendar days from date of 50% sprout emergence to the date that all plants in a plot senesced |
| 2 | Days to flowering | At flowering | number of days from planting to when 50% of the plants in a plot had at least one flower/inflorescence |
| 3 | Day to maturity | 5 months after planting | This was determined as the number of calendar days from the date of 50% sprout emergence to the date that all plants in a plot senesced. |
| 4 | Number of stems per plant | 2 months after emergence | Number of stems per plant was counted and recorded |
| 5 | Stem diameter | 5 months after emergence | measured using an electronic digital vernier caliper |
| 6 | Area under disease progression curve (YAD/YMV) | 2 months after planting | estimated using the trapezoidal method (Campbell and Madden, 1990)  $\mathrm{AUDPC}=\sum_{i=1}^{N} (\frac{y_{i+ y_{i+1}}}{2})(t_{i+1}-t_{i})$  where n = total number of observations, y_i_ = disease severity at the _i_th observation, and t = time at the _i_th observation.  The above formula was estimated from: Anthracnose severity was scored at two months after planting and thereafter, fortnightly as: 1 = no visible symptoms of anthracnose disease; 2 = few anthracnose spots or symptoms on 1 to 25% of the plant; 3 = anthracnose symptoms covering 26 to 50% of the plant; 4 = symptom on > 51% of the plant; 5 = severe necrosis and death of the plant. |
| 7 | Plant vigour | 2 months after emergence | Visual scoring as: 1= weak; 2= medium; 3 = vigorus |
| 8 | Plant Sex | at flowering | Visual scoring as: 0 = Not flowering (Unknown); 1 = Male; 2 = Female; 3= Monoecious male (m>f); 4= Monoecious female (f>m) |
| 9 | Flowering intensity | at flowering | visual examination (scoring) 0 = No bud; 1 = Aborted bud; 3 = low; 5 = Moderate; 7 = profuse; 9 = extremely profuse |
| 10 | Number of tubers per plant | after harvest | number of tubers harvested per plant were counted and classified into big size tubers (weighing 1 kg and above), medium size tubers (500g to 1 kg) and small size tubers (less than 500g) after weighing. |
| 11 | Tuber yield (kg plant^-1^) | after harvest | The weight of tubers harvested per plant was measured with a sensitive electronic weighing scale (AND HV-60KC, A&D company Ltd, Korea). |
| 12 | Tuber yield (t ha^-1^) | after harvest | weight of tubers harvested per plot measured with a sensitive electronic weighing scale (AND HV-60KC, A&D company Ltd, Korea) and classified as big size (above 1 kg), medium size (500g to 1 kg) and small size (less than 500g). The total tuber yield per plot was converted to tons per ha |
| 13 | Average tuber weight per plant | after harvest | Weight of tubers harvested per plot measured and divided by the total tuber number |
| 14 | Tuber appearance | after harvest | visual observation using a 1-4 scale where 1= rough; 2= Hairy; 3= thorny; 4= fully smooth |
| 15 | Spine on the Tuber | after harvest | pines on tuber surface:  The presence/intensity of thorns or spines on tuber surface was assessed using a visual scale of 0-7, where; 0= no thorns on tuber surface, 3= few thorns on tuber surface and 7= many thorns on tuber surface. |
| 16 | Tuber cracks | after harvest | Visual examination of tubers and rated as: 0= absent; 1 = few; 3 = many. |
| 17 | Tuber hairiness | after harvest | he presence/intensity of tuber hair or secondary roots on the surface of harvested tubers is recorded using a 1-3 scale where 1= No roots, 2= Few (few scattered roots in the surface at the proximal section of the tuber) and 3= Many roots on the entire surface of the tuber or many roots in one of the section of the tuber) |
| 18 | Canopy architecture | 2 months after emergence | predominant structural arrangement of branches and leaf distribution that gives a specific shape to the above ground part of the plant was assessed visually as: 1= One vine, palmiform, many branches and erect; 2= One vine 9palmiform erect, large and few branches; 3= Several vines, short, many branches, palmiform and erects; 4= One large vine, few branches, palmiform and prostrated |
| 19 | Leaf density | 5 months after emergence | Visual observation of variation in leaf mass as : 3= Low; 5= Intermediate; 7= High |
| 20 | Leaf shape | 5 months after emergence | Visual scoring as: 1= Ovate; 3 = Cordate; 5 = Sagittate;7 = Hastate |
| 21 | Senescence class | 6 months after emergence | foliage senescence of plants in a plot at 6 months after planting was scored using a scale of 1 to 9 where: 1 = very late; 3 = late; 5 = medium; 7 = early; 9 = very early |
| 22 | Spine on the stem | 5 months | Spines on stem of mature plant (5 months after emergence) was scored in a plot as: 0 = absent, 1 = few; 2 = many. |
| 23 | Inflorescence Type | From 2 month after planting | Score inflorescence type as 1 =Spike, 2 = Raceme; 3 = Panicle |
| 24 | Stem color | 2 month after planting | Visual assessment of the predominant color surface of stem on mature plant at five to six month after emergence using a 1-5 scale where 1= Green, 2= Purplish green, 3= Brownish green, 4= Dark brown and 5= Purple |
| 25 | Tuber length | after harvest | Tuber length measured with a flexible plastic measuring tape |
| 26 | Tuber width | after harvest | Tuber width measured with a flexible plastic measuring tape |
| 27 | Tuber area | after harvest | Length of tuber / tuber width |
| 28 | Tuber dry matter content (%) | after harvest | 200g of fresh tuber was chopped into small pieces and oven dried at 70°C till a constant weight was achieved. Percentage dry matter content was then be estimated as: $\frac{Tuber fresh weight-tuber dry weight}{tuber fresh weight}$ *100 |
| 29 | Tuber flesh oxidation | after harvest | Intensity of tuber flesh oxidation (degree of flesh surface colour change or browning of cut yam tuber) was assessed at different time intervals (0, 30, 60 and 180 minutes after cutting) using a 0-2 scale, where 0 = no oxidization, 1 = slightly oxidizing and 2 = highly oxidizing. |
